# Road damage detection algorithm for improved YOLOv5

**DOI:** 10.1038/s41598-022-19674-8

**Published:** 2022-09-15

**Authors:** Gege Guo, Zhenyu Zhang

**Affiliations:** 1 College of Software, Xinjiang University, Urumqi, 830046 China; 2 College of Information Science and Engineering, Xinjiang University, Urumqi, 830017 China; 3 Key Laboratory of Multilingual Information Technology in Xinjiang Uygur Autonomous Region, Xinjiang University, Urumqi, 830017 China

**Keywords:** Computational science, Computer science

## Abstract

Road damage detection is an important task to ensure road safety and realize the timely repair of road damage. The previous manual detection methods are low in efficiency and high in cost. To solve this problem, an improved YOLOv5 road damage detection algorithm, MN-YOLOv5, was proposed. We optimized the YOLOv5s model and chose a new backbone feature extraction network MobileNetV3 to replace the basic network of YOLOv5, which greatly reduced the number of parameters and GFLOPs of the model, and reduced the size of the model. At the same time, the coordinate attention lightweight attention module is introduced to help the network locate the target more accurately and improve the target detection accuracy. The KMeans clustering algorithm is used to filter the prior frame to make it more suitable for the dataset and to improve the detection accuracy. To improve the generalization ability of the model, a label smoothing algorithm is introduced. In addition, the structure reparameterization method is used to accelerate model reasoning. The experimental results show that the improved YOLOv5 model proposed in this paper can effectively identify pavement cracks. Compared with the original model, the mAP increased by 2.5%, the F1 score increased by 2.6%, and the model volume was smaller than that of YOLOv5. 1.62 times, the parameter was reduced by 1.66 times, and the GFLOPs were reduced by 1.69 times. This method can provide a reference for the automatic detection method of pavement cracks.

## Introduction

Cracks are common pavement distresses that seriously affect road safety and driving safety. For transportation agencies in most provinces and cities, maintaining high-quality road surfaces is one of the keys to maintaining road safety. The timely detection of pavement cracks is of great significance to prevent road damage and maintain traffic road safety. Traditional crack detection methods are mostly based on manual inspections or road survey vehicles equipped with various sensors. The efficiency of manual inspection is low, the risk factor is high, and the inspection results are affected by the subjective judgment of inspectors, which is not conducive to the accuracy rate. Road survey vehicles are expensive to build and can cost as much as $500,000^1^. Therefore, the research and application of fast, efficient, and accurate crack detection technology have great practical needs.

In recent years, with the rapid development of deep learning, object detection technology has also made remarkable achievements. Object detection technology is mainly divided into two categories. The first category is a region-based two-stage detection model, which is mainly divided into two processes. The first step is to propose several regions that may contain objects, and the second step is to run a classification network on the proposed regions to obtain the object category within each region. Common two-stage algorithms include fast region-based convolutional neural networks (Fast R-CNNs)^2^, region-based fully convolutional networks (R-FCNs)^3^, and mask region-based convolutional neural networks (Mask R-CNNs)^4^. The second type is a one-stage detection method based on regression, which directly separates specific categories and regresses the border. Its speed is faster than the two-stage detection method, but the accuracy is slightly lower. Common algorithms include the You Only Look Once^5,6^ series, Single Shot MultiBox Detector (SSD)^7^, and RetinaNet^8^.

Deep learning technology has also made great breakthroughs in the field of road damage detection. Naddaf-SH et al. ^9^ proposed using the one-stage network EfficientDet-D7^10^ to detect and classify asphalt pavement images and won the seventh place in the 2020 IEEE Big Data Challenge, but EfficientDet-D7 has the disadvantage of a large number of parameters. Hacıefendioğlu et al.^11^ used the two-stage network Faster R-CNN to detect concrete pavement cracks and studied the influence of different illumination and weather conditions on the model detection effect. Maeda et al.^12^ proposed using progressive growing of generative adversarial networks (PG-GANs)^13^ and Poisson blending methods to generate real road damage images as new training data to improve the accuracy of pothole detection. Mandal et al.^14^ proposed using the YOLO CSPDarknet53 network to detect road damage and won fourth place in the 2020 IEEE Big Data Challenge. Although the above research has made a certain contribution to the road damage detection task, there is still a large room for improvement in accuracy or detection speed. As one of the classic single-stage detection algorithms, the YOLO algorithm has been updated to YOLOv5, which has great advantages in detection accuracy and detection efficiency. Therefore, we choose to optimize the model based on YOLOv5s to further improve the accuracy of the model and reduce its size.

The main contributions of this study are as follows: (a) The real road environment is simulated. (b) For the specific task of pavement crack detection, we replace the backbone network of YOLOv5 and optimize the replaced backbone network combined with the attention mechanism to make it more suitable for the detection of this task. (c) In the optimized YOLOv5 model, we incorporate some other algorithms, such as structural reparameterization, the label smoothing algorithm, and the k-means algorithm, aiming to make our model more suitable for task-specific detection. (d) Compared with the unoptimized model, the model is improved to a certain extent, which verifies the superiority and effectiveness of the model in the field of pavement disease detection.

The rest of the paper is organized as follows: "Methodologies" describes in detail what we did, including the techniques used and improved methods. The "Datasets and evaluation parameters" section introduces and analyses our datasets and the metrics used to evaluate the strengths and weaknesses of our models. The "Analysis of Results" section presents the experimental results after deployment and discusses the strengths and weaknesses of our model. In the "Conclusion" section, we summarize the entire paper and propose ideas for further research improvements.

## Methodologies

### YOLOv5

YOLOv5 is a single-stage object detection model with four versions: YOLOv5s, YOLOv5m, YOLOv5l, and YOLOv5x. Among them, the fastest and smallest model is YOLOv5s, with a parameter of 7.0 M and a weight of 13.7 M. The algorithm framework is shown in the figure, which is mainly divided into three parts: the backbone network (Backbone), the bottleneck layer network (Neck), and the detection layer (Output). The backbone network consists of a focus module (Focus), standard convolution module (Conv), C3 module, and spatial pyramid pooling module (SPP).

**Figure 1:**
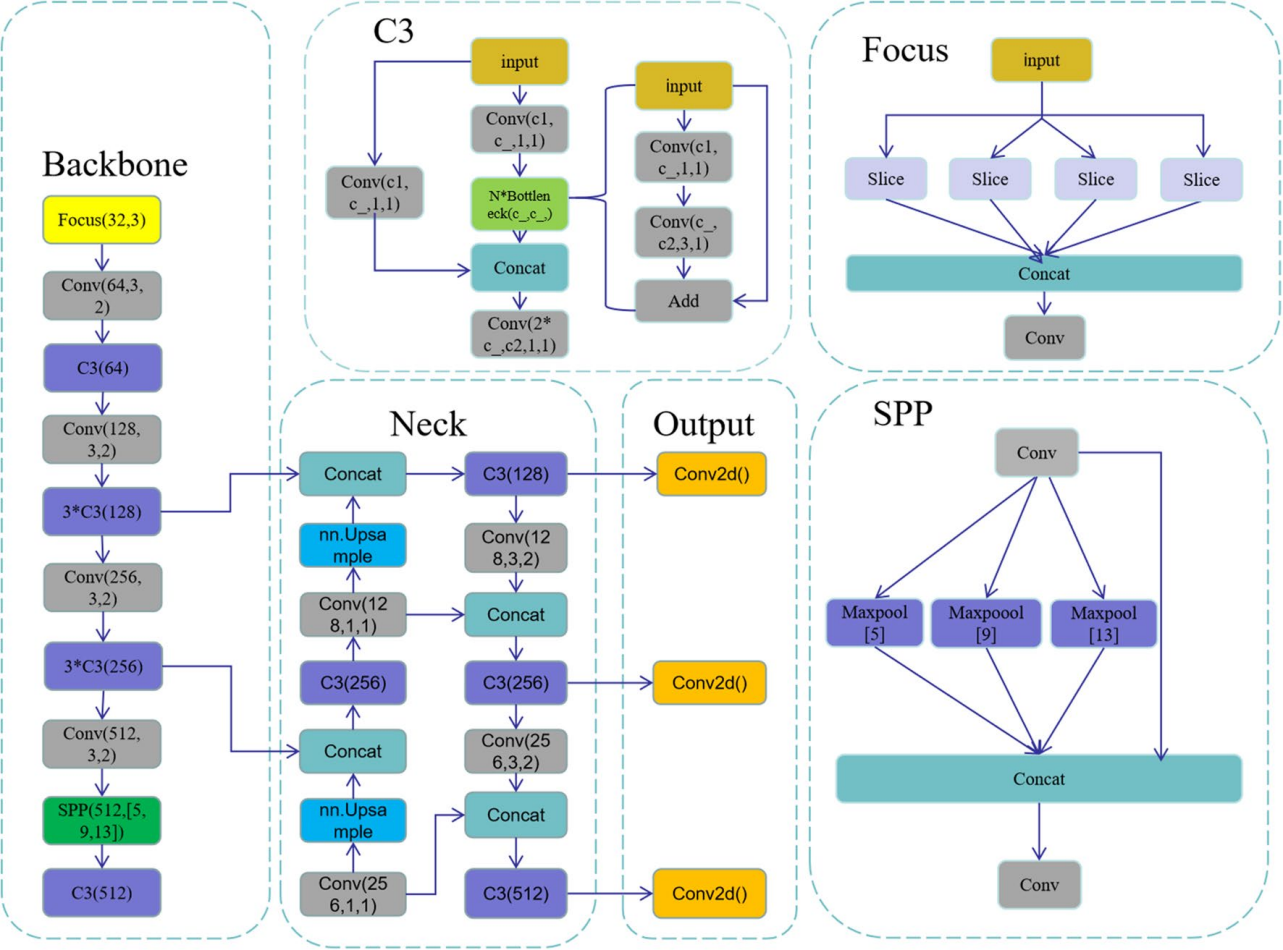
YOLOv5s structure.

In YOLOv5, the four versions of the network architecture are the same, and the size of the network structure is controlled by two parameters: depth factor (depth_multiple) and width factor (width_multiple). For example, the C3 operation in YOLOv5s is only done once, while the depth of YOLOv5l is three times that of v5s, so three C3 operations will be performed. The specific network structure of the algorithm is shown in Fig. 1.

### Improved YOLOv5

**Figure 2:**
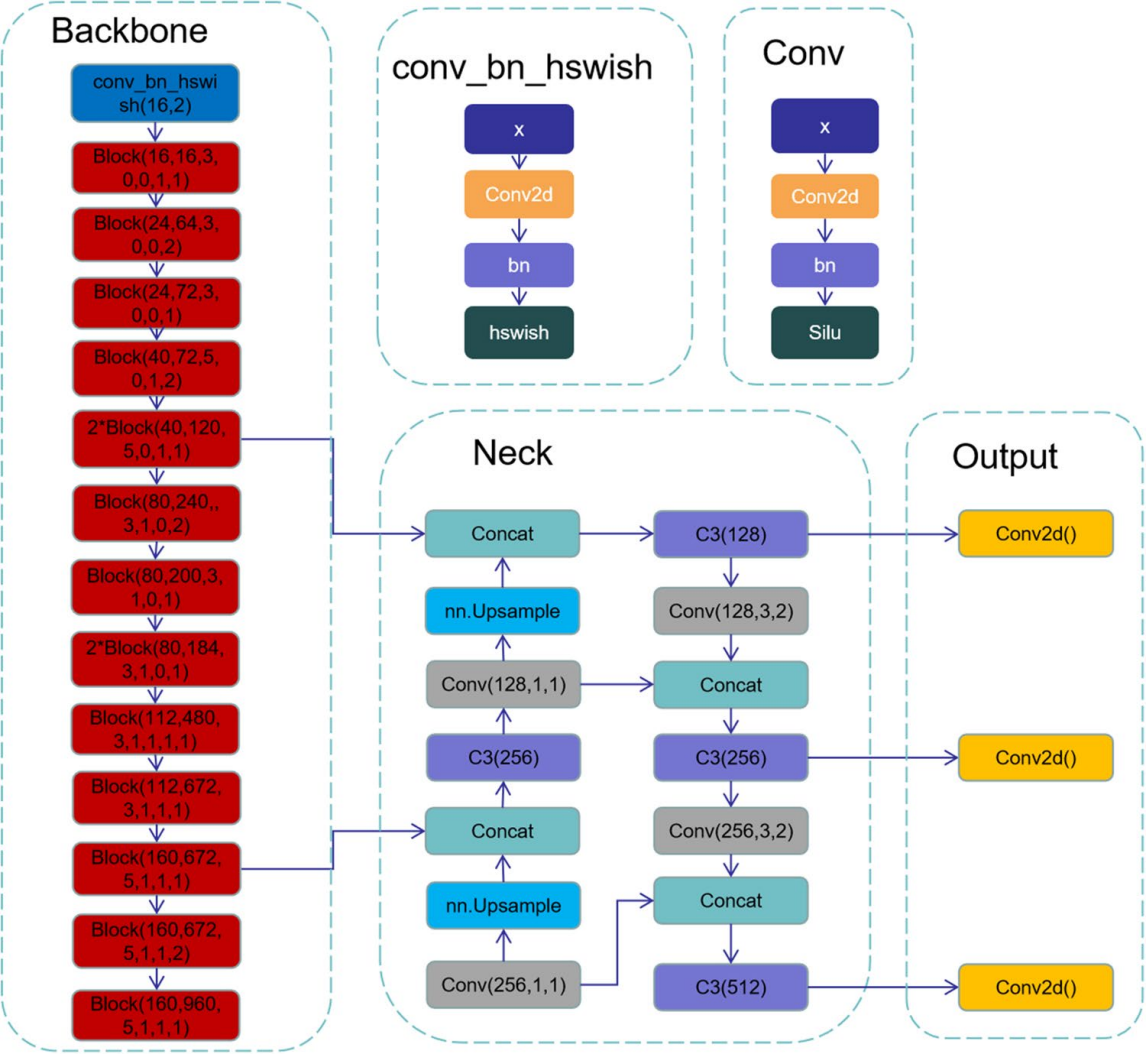
Experimental model diagram.

Since the YOLOv5s algorithm uses multilayer feature map prediction and is a one-stage network, it has good results in terms of accuracy and detection speed. Especially in terms of speed, it meets the requirements of pavement crack detection tasks and can be well deployed in industry. This paper attempts to improve the YOLOv5s model, reduce the size of the model, and improve the detection accuracy by using a lightweight network structure in terms of accuracy, parameter amount, and calculation amount while ensuring accuracy. First, by comparing various convolutional structures, MobileNetV3 is finally selected as the backbone network of the model. Second, the method of structural reparameterization is adopted to fuse the model convolution layer and the batch normalization (BN) layer to optimize the model inference speed. At the same time, we conducted research and analysis on the pavement crack dataset and found that different crack types are similar and easy to confuse, which may lead to poor model generalization ability. Therefore, this paper presents a label smoothing technique for solving this problem. To improve the accuracy of the model, we introduced the Coordination Attention module to improve the backbone network of the model. Finally, because the YOLOv5 model uses the a priori frame mechanism and the artificial experience design of the a priori frame is too subjective, the KMeans algorithm is used to iteratively analyse the dataset, and 9 a priori frames suitable for the dataset in this article are selected to improve the accuracy. The specific network structure of the algorithm is shown in Fig. 2.

### Selection of the backbone network

In 2019, Howard et al. proposed the MobileNetV3^15^ network. As the latest version of the MobileNet series, it has the characteristics of small parameters, high accuracy, and fast real-time detection speed and is widely used in embedded and mobile terminals. The MobileNetV3 network inherits the features of MobileNetV1's depthwise separable convolution and MobileNetV2's inverse residual structure with a linear bottleneck and uses the NetAdapt algorithm to search and optimize the number of convolution kernels and channels. To reduce the number of parameters and GFLOPs of the model, this paper uses MobileNetV3 as the backbone network for feature extraction. The contents of MobileNetV3 are as follows:

#### Depthwise separable convolution

The depthwise separable convolution^16^ can be divided into two parts: depthwise convolution and point convolution. The depthwise convolution adopts different convolution kernels for each input channel, that is, the number of groups of the network is the same as the number of channels of the network, thereby reducing the calculation amount of convolution and then using point convolution to fuse between channels. Assuming that $D_{K} \times D_{K}$ is the size of the convolution kernel, $M$ is the number of input channels, $N$ is the number of output channels, and $D_{F} \times D_{F}$ is the size of the output feature map, then the calculation amount of ordinary convolution is shown in formula (1):

1 $$D_{K} \times D_{K} \times M \times N \times D_{F} \times D_{F}$$

The calculation amount of the depthwise separable convolution is shown in formula (2):

2 $$D_{K} \times D_{K} \times M \times D_{F} \times D_{F} + M \times N \times D_{F} \times D_{F}$$

As shown in Eq. (3), the depthwise separable convolution is equivalent to compressing the calculation amount of ordinary convolution as:

3 $$\frac{{D_{K} \times D_{K} \times M \times D_{F} \times D_{F} + M \times N \times D_{F} \times D_{F} }}{{D_{K} \times D_{K} \times M \times N \times D_{F} \times D_{F} }} = \frac{1}{N} + \frac{1}{{D_{K}^{2} }}$$

With the depthwise separable convolution, the amount of computation is greatly reduced.

#### Inverse residual structure with linear bottlenecks

**Figure 3:**
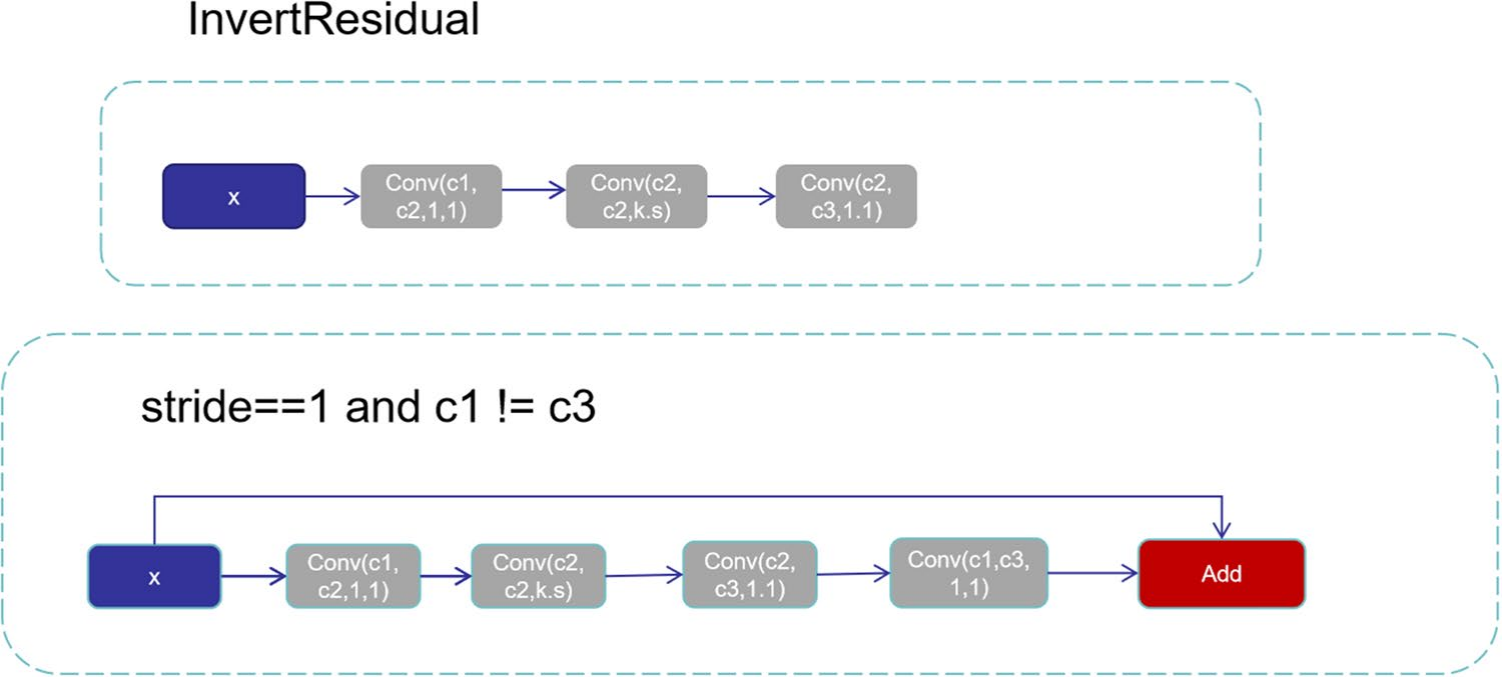
InvertResidual structure.

Since the activation function ReLU used by the original residual block has less available information in low dimensions, it easily causes information loss, so MobileNetV2^17^ proposed an inverted residual structure with a linear bottleneck. The original residual structure adopts the method of reducing the dimension first and then increasing the dimension, but the depth convolution parameters are few, and the extracted features are relatively small. Therefore, the inverted Resblock first expands to perform feature extraction and then compresses, and the inverted Resblock first uses a 1 × 1 convolution to increase the dimension, then reduces the number of calculation parameters through the 3 × 3 depthwise separable convolution (DWConv), then reduces the dimension through a 1 × 1 convolution, and finally connects the result to the input. In addition, after the convolutional layer has performed dimensionality reduction, activation functions such as ReLU are no longer added for nonlinear transformation. The purpose of this is to avoid information loss as much as possible. As shown in Fig. 3.

#### Squeeze-and-excitation networks

**Figure 4:**
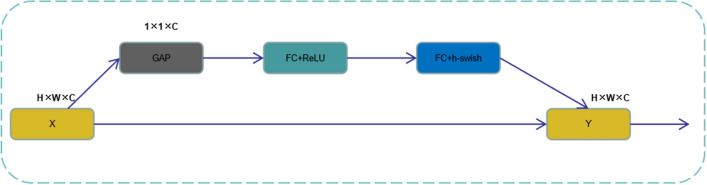
Squeeze-and-excitation networks.

Squeeze-and-excitation networks (SENet)^18^ is a channel attention mechanism proposed by Hu et al. at CVPR in 2018. Its core idea is to model the interdependence between channels and generate corresponding weights for each channel to improve important features. and suppress unimportant features. The process of the SENet network is divided into two steps: squeeze and excitation. Squeeze obtains the global compressed feature vector of the current feature map by performing the global average pooling (GAP) operation on the extracted features. Excitation obtains the weight of each channel after normalization through two layers of full connection, and the weighted features are used as the input to the next layer of the network. The SENet attention mechanism structure is shown in Fig. 4.

The size of input $X$ is $H \times W \times C$, $GAP$ represents global average pooling, FC represents a fully connected layer, ${\text{Re}} LU$ and $h - swish$ are activation functions, and $Y$ multiplies the generated weight coefficient of each channel with all elements of the corresponding channel. The important features are enhanced, and the unimportant features are weakened so that the extracted features are more directional.

#### h-swish

The activation function $h{\text{-}}swish$ is improved based on $swish$, replacing the original $sigmoid$ function with ${\text{Re}} LU6(x + 3)/6$. The swish function has the characteristics of no upper bound, lower bound, smoothness, and nonmonotonicity, and it is better than ReLU in deep models. However, because the $sigmoid$ function is complex to calculate and consumes many resources on the mobile side, MobileNetV3 uses the approximate function ${\text{Re}} LU6(x + 3)/6$ to approximate swish. This replacement reduces problems such as the disappearance of network gradients due to the increase in the number of network layers and can also effectively reduce the amount of computation, improve model performance, and improve model detection efficiency. 15% efficiency.

### Improvement of the backbone network

**Figure 5:**
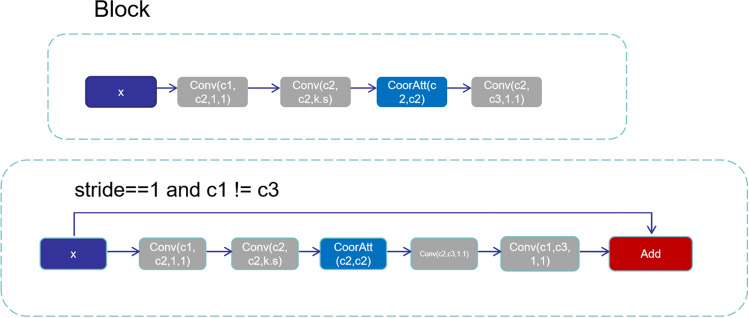
Block structure.

The optimization of the feature extraction network is based on the needs of the road damage detection algorithm, which further improves the detection and identification of the four types of pavement cracks in the model, making it more suitable for pavement crack detection tasks. The MobileNetV3 network consists of an inverted residual with linear bottleneck modules, which greatly reduces the number of parameters and GFLOPs of the model, but there is still room for improvement in detection accuracy. Therefore, we added the attention mechanism coordinate attention module to improve the inverted residual with a linear bottleneck module in this study to more fully improve the detection accuracy of the model for specific tasks. The improved module is named Block, as shown in Fig. 5:

#### Coordinate attention

**Figure 6:**
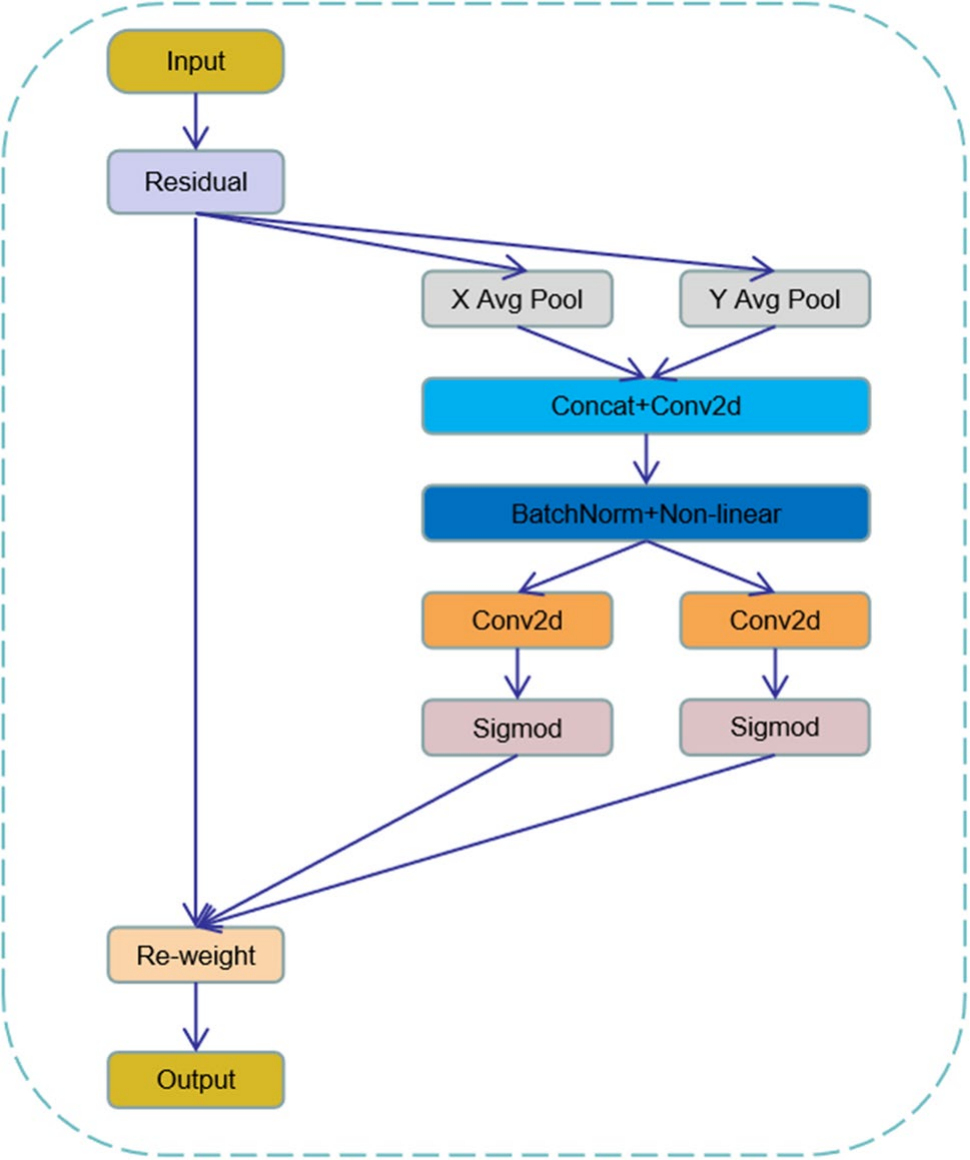
CA structure.

In CVPR2021, Hou et al. proposed the coordinate attention^19^ module, which is a new attention module proposed for channel attention that ignores location information that is important for generating spatially selective attention. $CA$ encodes channel relations and long-term dependence through precise location information, and the specific operation is divided into two steps: embed coordinate information and coordinate attention generated. Its structure is shown in Fig. 6.

#### Embedding of coordinate information

Global pooling is usually used for global encoding of channel attention encoding spatial information, but it is difficult to save location information because it compresses global spatial information into channel descriptors. To enable the attention module to capture remote spatial interactions with precise location information, this paper decomposes global pooling into a pair of one-dimensional feature coding operations according to the following formula:

4 $$Z_{c} = \frac{1}{H \times W}\sum\limits_{i = 1}^{H} {\sum\limits_{j = 1}^{W} {x_{c} (i,j)} }$$

Specifically, given input, the pooling kernel of size $(H,1)$ or $(1,W)$ is first used to encode each channel along with horizontal and vertical coordinates, respectively. Therefore, the output of the first channel whose height is can be expressed as:

5 $$Z_{c}^{h} (h) = \frac{1}{W}\sum\limits_{0 \le i \le W} {x_{c} (h,i)}$$

Similarly, the output of the first channel of width can be written as:

6 $$Z_{c}^{w} (w) = \frac{1}{H}\sum\limits_{0 \le j \le H} {x_{c} (j,w)}$$

The above two transformations aggregate features along two spatial directions to obtain a pair of direction-aware feature maps. This is very different from SENet, which produces a single eigenvector in the channel attention approach. These two transformations also allow the attention module to capture long-term dependencies along one spatial direction and store precise location information along the other spatial direction, which helps the network more accurately locate targets of interest.

#### Generation of coordinated attention

Coordinate information embedding can well obtain the global receptive field and encode accurate location information through the above transformation. To utilize the resulting representation, the author proposed a second transformation called coordinate attention generation. After passing the transformation in information embedding, this part concatenates the above transformation and then uses the convolution transformation function to transform it:

7 $$f = \delta (F_{1} ([z^{h} ,z^{w} ]))$$

where the concatenation operation along a spatial dimension is the nonlinear activation function and is the intermediate feature mapping encoding spatial information in horizontal and vertical directions. Here, it is used to control the reduction rate of the SE block size. Then, along the dimension of space, it will decompose into two separate tensors. They are transformed into tensors with the same number of channels as the input by using the other two convolution transformations to obtain:

8 $$g^{h} = \sigma (F_{h} (f^{h} ))$$

9 $$g^{w} = \sigma (F_{w} (f^{w} ))$$

Here is the $sigmoid$ activation function. To reduce model complexity and computational overhead, an appropriate reduction ratio is usually used to reduce the number of channels. The output is then expanded as attention weights. Finally, the output of the coordinate attention block can be written as:

10 $$y_{c} (i,j) = x_{c} (i,j) \times g_{c}^{h} (i) \times g_{c}^{w} (j)$$

### Auto anchor searching

In object detection, the model needs to learn not only the category of the object but also the location and size of the object. Since there are objects of different scales and aspect ratios in each image, it is difficult for the model to learn the shapes of different objects during the training process. Therefore, Ren et al.^20^ proposed an a priori box mechanism in Faster R-CNN to solve this problem. The a priori box mechanism divides the space where objects with different scales and aspect ratios are located into several subspaces, which reduces the difficulty of the problem and the difficulty of model learning. This mechanism is also widely used in various excellent object detection models, such as SSD, YOLOv3, and RetinaNet. The artificially designed a priori boxes are highly subjective and cannot guarantee that they will fit the dataset well. Therefore, by analysing the shape and characteristics of pavement cracks, this paper uses the KMeans algorithm to cluster the training set to obtain 9 a priori boxes as the initial clustering boxes. The core goal of the KMeans clustering algorithm is to divide the dataset into K clusters, give the corresponding center point of each sample data, and automatically generate a set of a priori boxes that are more suitable for the dataset, thereby effectively reducing the initial cost of the original algorithm. According to the clustering deviation caused by clustering points, an a priori frame of suitable size is obtained and matched to the corresponding feature map, which effectively improves the detection accuracy and recall rate.

The initial anchor boxes of the YOLOv5 model are '10, 13, 16, 30, 33, 23', '30, 61, 62, 45, 59, 119' and '116, 90, 156, 198, 373, 326', which are used to detect small, medium, large objects. The original anchor box is only suitable for the COCO dataset, not for the pavement crack data. We use the KMeans algorithm to recluster the pavement crack dataset to obtain a new anchor box suitable for our dataset. The new anchor box is '46, 29, 104, 23, 39,66', '86,75, 206,36, 91,149', '168,107, 221,216, 455,249'.

### Label smoothing

Label smoothing is an effective regularization strategy for deep neural networks, and it is often used to reduce the overfitting problem of training deep neural networks (DNNs) and further improve classification performance. Overfitting is a common problem encountered in the training of deep learning models, which means that the model performs well in the training set, but the performance in the test set is not satisfactory, the generalization is poor, and the model cannot be effectively used to predict unknown data. Neural network training will prompt itself to learn in the direction with the largest difference between the correct label and the wrong label. When the training data are small and insufficient to represent all the sample features, it is easy to cause the network to overfit.

Label smoothing solves the above problems through the following ideas. During training, it is assumed that there are incorrectly labelled labels to avoid overconfidence in the labels of training samples. Taking the classification problem as an example, we usually think that the object category probability in the training data is 1, the nonobject probability is 0, and the traditional label vector ${\text{y}}_{{\text{i}}}$ is

11 $${\text{y}}_{{\text{i}}} = \left\{ {\begin{array}{*{20}c} {1,} & {i = target} \\ {0,} & {i \ne target} \\ \end{array} } \right.$$

Label smoothing combines a uniform distribution, replacing the traditional label vector ${\text{y}}_{{\text{i}}}$ with the updated label vector $\hat{y}_{{_{i} }}$:

12 $$\hat{y}_{{_{i} }} = y_{{\text{i}}} (1 - \partial ) + \partial /K$$

where is the total number of $K$ multicategory categories and $\partial$ is a small hyperparameter (usually 0.1), that is,

13 $${\hat{\text{y}}}_{{\text{i}}} = \left\{ {\begin{array}{*{20}c} {1 - \partial ,} & {i = target} \\ {\partial /K,} & {i \ne target} \\ \end{array} } \right.$$

In this way, the smoothed distribution of the labels is equivalent to adding noise to the real distribution, preventing the model from being overconfident in the correct label, reducing the difference between the output values of predicted positive and negative samples, thereby avoiding overfitting and improving the generalization of the ability of the model.

In this study, with a $\partial$ set of 0.1, the label smoothing method can significantly improve the performance of the model when the training set is not too large.

### Structural reparameterization

The concept of structural reparameterization refers to constructing a series of network structures in the training phase and transforming the network structure into another set of network structures by equivalently transforming its parameters into another set of parameters in the inference phase. The advantage of this method lies in that we can use the network structure with a larger structure and certain good properties, such as higher accuracy and sparsity, to train the dataset during training, while the converted reasoning structure is smaller and retains such properties, thus obtaining a more efficient and convenient deployment model.

Ding Xiaohan implemented this idea in the RepVGG^21^ network structure proposed in ICCV2021. The method of mathematical derivation is mainly used in the idea of structural reparameterization, and the validity of the idea is verified by experiments. This paper mainly adopts the idea of Conv-BN merging. In neural network training, the BN layer can speed up network convergence, suppress overfitting, and effectively solve the problems of gradient disappearance and gradient explosion. However, when the network infers forward, the BN layer will occupy more memory and video memory, which will affect the performance of the model. Therefore, it is necessary to use the fusion of the Conv layer and BN layer to speed up the inference speed of the network. The formula derivation is as follows: The formula is calculated by the convolution layer, as shown in formula 14, where $\upomega$ is the weight and $b$ is the bias:

14 $$X_{1} =\upomega x + b$$

The calculation of the BN layer is shown in formula 15, where $\upgamma$ and $\upbeta$ are learning parameters, $u$ is the sample mean, $\sigma$ is the variance, and $\varepsilon$ represents a small number (preventing the denominator from being 0):

15 $$X_{2} =\upgamma \frac{{X_{1} - u}}{{\sqrt {\upsigma ^{2} +\upvarepsilon } }} +\upbeta$$

Formula 14 is substituted into formula 15, and convolution and BN are combined to obtain formula 16:

16 $$X_{2} =\upgamma \frac{{\upomega x + b - u}}{{\sqrt {\upsigma ^{2} +\upvarepsilon } }} +\upbeta$$

Equation 17 can be obtained by splitting Eq. (16) as follows:

17 $$X_{2} =\upgamma \frac{{\upgamma \upomega }}{{\sqrt {\upsigma ^{2} +\upvarepsilon } }}x + {\upbeta + \gamma }\frac{b - u}{{\sqrt {\upsigma ^{2} +\upvarepsilon } }}$$

Let $\frac{{\upgamma \upomega }}{{\sqrt {\upsigma ^{2} +\upvarepsilon } }} = {\upomega ^{\prime}}$, ${\upbeta + \gamma }\frac{b - u}{{\sqrt {\upsigma ^{2} +\upvarepsilon } }} = b^{\prime}$, obtain formula 18:

It can be seen that the final merged convolution is still composed of weight w and bias b.

18 $$X_{2} = {\upomega ^{\prime}}x + b^{\prime}$$

According to the homogeneity of convolution, the process of merging BN layers is a linear operation, which is equivalent to modifying the convolution kernel, while the convolution remains unchanged. Therefore, according to the reasoning, the combination of the convolutional layer and the BN layer can improve the speed of the forward inference of the model.

## Datasets and evaluation parameters

### RDD2020

The dataset used in this paper is the open-source dataset RDD2020^22^, which consists of road images from three countries: Japan, India, and the Czech Republic. The dataset includes longitudinal cracks D00, transverse cracks D10, mesh cracks D20, pothole D40, longitudinal construction joint part D01, lateral construction joint part D11, crosswalk blur D43, and white line blur D44. The first four types of cracks are the main types of road damage. Therefore, in this study, the damage categories considered are longitudinal cracks D00, transverse cracks D10, network cracks D20, and pothole D40. The dataset is divided into the training set, test set 1, and test set 2. The training set has a total of 21,041 images, and test set 1 and test set 2 have 2631 images and 2664 images, respectively. Since some pictures in the training set do not contain the detection target of this study, it is necessary to process the training set and screen out the pictures that do not contain the detection target of the research. After analysis and processing, among the 10,506 pictures from Japan, 7900 pictures contain the detection target of this research, and among the 2829 pictures from the Czech Republic, 1072 pictures contain the detection target of this research. Among the 7706 pictures from India, there are 3223 images containing the detection target of this study, so there are a total of 12,195 training set images with detection target instances. After filtering out images that do not contain the four crack types detected in the study, we randomly select 1211 images from the training set as the validation set according to the 1:9 partitioning rule. The images in the training set are preannotated with different categories of road damage as ground-truth labels. The test set does not contain real labels, and the predicted value file needs to be output and uploaded to the IEEE Big Data 2020 website to obtain the F1 score. Table 1 lists all four types of road damage, namely, longitudinal cracks, transverse cracks, network cracks, and pothole.

**Table 1:**
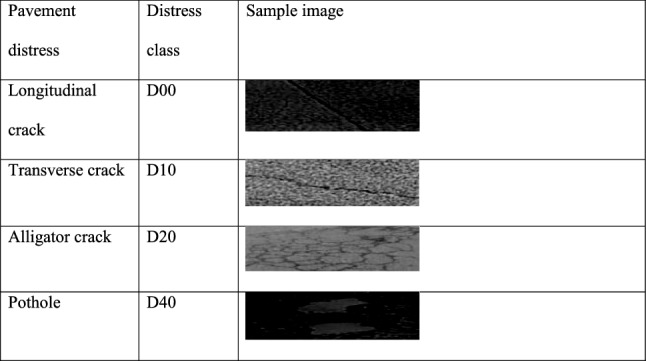
Dataset category.

### Evaluation parameters

The environment for this experiment is the Ubuntu18.04 operating system, the model algorithm is implemented through the PyTorch deep learning framework, the graphics card is an NVIDIA GeForce RTX 2080 Ti, the CPU is an Intel XeonE5-2678v3, and the memory is 62 GB.

During training, the input image is set to 640 × 640, and SGD is used as the optimization function to train the model. The model training period (epochs) is 100, the batch size is 24 and the initial learning rate is 0.02. This experiment adopts the same data augmentation algorithm as the original YOLOv5 algorithm.

The evaluation indicators used in this paper are precision, recall rate (recall), F1 score, mean Average Precision (mAP), the number of parameters (Params), Giga Floating-point Operations Per Second (GFLOPs), and Frames Per Second (FPS). Among them, the precision and recall rate are used as the basic indicators, and the F1 score and mAP calculated according to the precision and recall rate are used as the final evaluation indicators to measure the recognition accuracy of the model. GFLOPs are used to measure the complexity of the model or algorithm, and Params represents the size of the model. Usually, the smaller the Params and GFLOPs are, the smaller the computing power required to represent the model, the lower the performance requirements for hardware, and the easier it is to build in low-end devices.

19 $$precision = \frac{tp}{{tp + fp}}$$

20 $$recall = \frac{tp}{{tp + fn}}$$

21 $$F1 = 2\,\frac{precision \times recall}{{precision + recall}}$$

## Results and analysis

### Evaluation results

To verify the effectiveness of the improved YOLOv5 algorithm in the road damage detection task, five groups of experiments are designed in this paper. Experiment 1 compares the performance of the original YOLOv5s algorithm and MobileNetV3 as the backbone network. Since the default learning rate of the original YOLOv5 algorithm is 0.01, and the learning rate of the improved model is 0.02, for a fairer comparison, the experiments show the performance of the original YOLOv5s algorithm under different learning rates. Experiments show that when using MobileNetV3 as the backbone network, compared with the original YOLOv5 algorithm with learning rates of 0.01 and 0.02, the F1 score is increased by 1.4% and 0.5%, and the mAP is increased by 1.6% and 1.1%. At the same time, the number of GFLOPs and parameters was greatly reduced, as shown in Table 2. (LR is the learning rate.)

**Table 2:** Performance comparison of MobileNetV3.

Model	Params/M	GFLOPs	Precision (%)	Recall (%)	mAP (%)	F1 (%)	FPS
YOLOv5s-LR = 0.01	7.0	16.4	56.5	50.4	50.6	53.1	67
YOLOv5s-LR = 0.02	7.0	16.4	55.6	52.4	51.1	54.0	67
MobileNetV3-YOLOv5	4.0	9.3	55.1	53.8	52.2	54.5	60

To further improve the model performance, this paper introduces the KMeans clustering algorithm to replace the prior box. Experiment 2 compared the effect of using the original YOLOv5s algorithm a priori frame and the a priori frame obtained by using the KMeans clustering algorithm, and the effect is shown in the table. Compared with the original a priori frame, the a priori frame selected by the KMeans algorithm has a 0.7% increase in mAP and a 0.4% increase in the F1 score. As shown in Table 3.

**Table 3:** Performance comparison of KMeans.

Model	Params/M	GFLOPs	Precision (%)	Recall (%)	Map (%)	F1 (%)	FPS
MobileNetV3-YOLOv5	4.0	9.3	55.1	53.8	52.2	54.5	60
MobileNetV3-YOLOv5 + KMeans	4.0	9.3	57.2	52.7	52.9	54.9	60

To accurately locate the object position, the CA module is introduced in this paper, and the third experiment is the model after integrating the CA module. Compared with the unused model, the experiment found that the introduction of the CA module increased mAP by 0.4%, and the F1 score increased by 1.0%, as shown in Table 4.

**Table 4:** Performance comparison of CA.

Model	Params/M	GFLOPs	Precision (%)	Recall (%)	mAP (%)	F1 (%)	FPS
MobileNetV3-YOLOv5 + KMeans	4.0	9.3	57.2	52.7	52.9	54.9	60
MobileNetV3-YOLOv5 + KMeans + CA	4.2	9.7	58.4	53.7	53.3	55.9	36

Since road damage detection is usually used in industry, to meet the lightweight requirements and facilitate the deployment of the model on mobile terminals or embedded terminals, this paper adopts the fusion method of the structurally reparameterized BN layer and the convolution layer. The fourth experiment is to verify the effect of structural reparameterization. The experiment found that the FPS of the model using structural reparameterization was increased by 15% compared with the model without structural reparameterization, which effectively accelerated the model inference speed, as shown in Table 5.

**Table 5:** Comparison of the performance of the structural reparameterization.

Model	Params/M	GFLOPs	Precision (%)	Recall (%)	mAP (%)	F1 (%)	FPS/
MobileNetV3-YOLOv5 + KMeans + CA	4.2	9.7	58.4	53.7	53.3	55.9	36
MobileNetV3-YOLOv5 + KMeans + CA + Conv-BN	4.2	9.7	58.4	53.7	53.3	55.9	42

Due to the imbalance of the dataset categories, this paper introduces the label smoothing algorithm, and the fifth experiment verifies the effect of the label smoothing algorithm. Experiments show that the model using the label smoothing algorithm has a 0.3% increase in mAP and a 0.7% increase in F1 score compared to the unused model, as shown in Table 6.

**Table 6:** Performance comparison of LabelSmoothing.

Model	Params/M	GFLOPs	Precision (%)	Recall (%)	mAP (%)	F1 (%)	FPS
MobileNetV3-YOLOv5 + KMeans + CA + Conv-BN	4.2	9.7	58.4	53.7	53.3	55.9	42
MobileNetV3-YOLOv5 + KMeans + CA + Conv-BN + LabelSmoothing	4.2	9.7	57.2	56	53.6	56.6	42

### Comparison of detection results of different algorithms

It can be seen from the experiments that the algorithm proposed in this paper can achieve an F1 score of 56.6% and a mAP value of 53.6% on the verification set of the road damage detection task. Compared with the original YOLOv5s algorithm, the mAP is increased by 2.5%, and the F1 score is increased by 2.6%. An F1 score of 61.86% and an F1 score of 60.92 were achieved on the two test sets, and the comparison with the results submitted on the Global Road Damage Detection Challenge'2020 is shown in Table 7^10,15,23–25^.

**Table 7:** Comparison of the detection results of different algorithms.

Model	Test1-F1 (%)	Test2-F1 (%)
YOLOv5x	56.83	57.10
Ensemble(YOLO-v4 + Faster-RCNN)	56.36	57.07
EfficientDet	56.5	54.7
YOLOv4	55.4	54.1
YOLO model trained on CSPDarknet53 backbone	58.14	57.51
Multi-stage Faster R-CNN with Resnet-50 and Resnet-101 backbones	53.68	54.26
Road Damage Detector using Detectron2 and Faster R-CNN	51	51.4
FR-CNN; Classifying the region and using regional experts for the detection	47.20	46.56
Ours	61.86	60.92

At the same time, the parameter amount and calculation amount of the algorithm is small, the GFLOPs is 9.7, and the parameter amount is 4.2 M. Compared with the GFLOPs 16.4 and 7.0 M parameters of the original YOLOv5s algorithm, the algorithm proposed in this paper has greater improvement and better performance.

**Figure 7:**
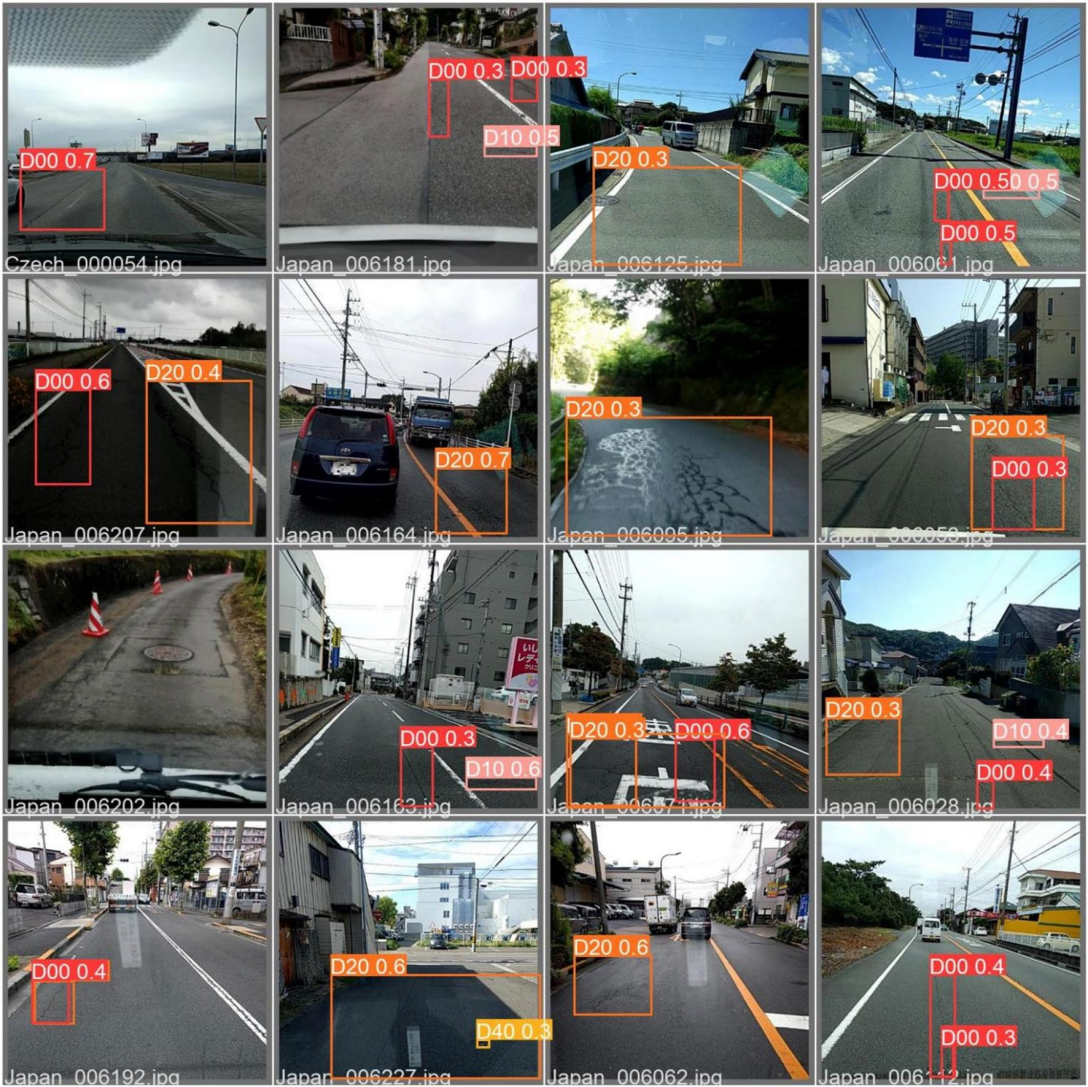
Inspection effect picture.

This paper fuses the MobileNetV3 network with the CA module, uses the KMeans algorithm and the Label-Smoothing algorithm and fuses the convolutional layer and the BN layer. The experiment verifies the effectiveness of the algorithm, and Fig. 7 shows the detection effect of the algorithm.

## Conclusion

This paper proposes an improved road damage detection method based on the YOLOv5s model, which uses a large amount of data to train the network model to detect various pavement cracks. In the proposed method, first, to reduce the size of the model, we propose to use the lightweight network MobileNetV3 as the backbone network to replace the backbone network of the original YOLOv5s model to reduce the number of parameters and GFLOPs of the model. Then we use the Coordinate Attention module to optimize the MobileNetV3 network to improve the model detection accuracy. Second, we use the KMeans clustering algorithm to select three sets of anchor boxes for our model that are more suitable for the pavement crack detection task, which improves the model detection accuracy and uses the LabelSmoothing algorithm to enhance the generalization ability of the model. Finally, we integrate the BN layers and convolutional layers of the model to improve the model inference speed. Experiments show that compared with the original YOLOv5s, our model improves the accuracy, reduces the number of parameters and GFLOPs, and is suitable for scenarios with accuracy requirements and limited memory and computing power, such as embedded devices. In the future, we will further optimize the network model to improve its accuracy, so that the model can be better applied to the pavement crack detection task.

## Data Availability

Data or code presented in this study is available on request from the corresponding author.
